# HER2-Specific Pseudomonas Exotoxin A PE25 Based Fusions: Influence of Targeting Domain on Target Binding, Toxicity, and In Vivo Biodistribution

**DOI:** 10.3390/pharmaceutics12040391

**Published:** 2020-04-24

**Authors:** Haozhong Ding, Mohamed Altai, Wen Yin, Sarah Lindbo, Hao Liu, Javad Garousi, Tianqi Xu, Anna Orlova, Vladimir Tolmachev, Sophia Hober, Torbjörn Gräslund

**Affiliations:** 1 Department of Protein Science, KTH Royal Institute of Technology, Roslagstullsbacken 21, 114 17 Stockholm, Sweden; haozhong@kth.se (H.D.); wenyin@kth.se (W.Y.); slindbo@kth.se (S.L.); haoliu2@kth.se (H.L.); sophia@kth.se (S.H.); 2 Department of Oncology and Pathology, Barngatan 4, Lund University, 222 42 Lund, Sweden; mohamed.altai@med.lu.se; 3 Department of Immunology, Genetics and Pathology, Dag Hammarskjölds väg 20, Uppsala University, 751 85 Uppsala, Sweden; javad.garousi@igp.uu.se (J.G.); tianqi.xu@igp.uu.se (T.X.); vladimir.tolmachev@igp.uu.se (V.T.); 4 Department of Medicinal Chemistry, Dag Hammarskjölds väg 14C; Uppsala University, 751 23 Uppsala, Sweden; anna.orlova@ilk.uu.se; 5 Research Centrum for Oncotheranostics, Research School of Chemistry and Applied Biomedical Sciences, Tomsk Polytechnic University, 634050 Tomsk, Russia

**Keywords:** pseudomonas exotoxin A, affibody molecule, half-life extension, cancer, HER2

## Abstract

The human epidermal growth factor receptor 2 (HER2) is a clinically validated target for cancer therapy, and targeted therapies are often used in regimens for patients with a high HER2 expression level. Despite the success of current drugs, a number of patients succumb to their disease, which motivates development of novel drugs with other modes of action. We have previously shown that an albumin binding domain-derived affinity protein with specific affinity for HER2, ADAPT_6_, can be used to deliver the highly cytotoxic protein domain PE25, a derivative of Pseudomonas exotoxin A, to HER2 overexpressing malignant cells, leading to potent and specific cell killing. In this study we expanded the investigation for an optimal targeting domain and constructed two fusion toxins where a HER2-binding affibody molecule, Z_HER2:2891_, or the dual-HER2-binding hybrid Z_HER2:2891_-ADAPT_6_ were used for cancer cell targeting. We found that both targeting domains conferred strong binding to HER2; both to the purified extracellular domain and to the HER2 overexpressing cell line SKOV3. This resulted in fusion toxins with high cytotoxic potency toward cell lines with high expression levels of HER2, with EC_50_ values between 10 and 100 pM. For extension of the plasma half-life, an albumin binding domain was also included. Intravenous injection of the fusion toxins into mice showed a profound influence of the targeting domain on biodistribution. Compared to previous results, with ADAPT_6_ as targeting domain, Z_HER2:2891_ gave rise to further extension of the plasma half-life and also shifted the clearance route of the fusion toxin from the liver to the kidneys. Collectively, the results show that the targeting domain has a major impact on uptake of PE25-based fusion toxins in different organs. The results also show that PE25-based fusion toxins with high affinity to HER2 do not necessarily increase the cytotoxicity beyond a certain point in affinity. In conclusion, Z_HER2:2891_ has the most favorable characteristics as targeting domain for PE25.

## 1. Introduction

Therapeutic approaches targeting particular pathways or molecular abnormalities on cancer cells have shown great progress during recent years and have led to impressive treatment responses in some cases. Overexpression of human epidermal growth factor receptor 2 (HER2) is a molecular abnormality that has attracted a lot of attention [1]. It is overexpressed in a significant number (approximately 25%) of all breast cancers and to a lesser extent also in other cancer types [2]. HER2 is a single-pass plasma membrane anchored receptor that belongs to the epidermal growth factor receptor family. It can form homo- or hetero-dimers with other members of its family to trigger activation of e.g., the mitogen activated protein kinase (MAPK) and PI3K/Akt pathways [3]. This leads to a more aggressive phenotype which is characterized by increased proliferation, survival, and motility, and a poorer prognosis for the patient [4].

Several HER2-specific drugs have been approved by the US Food and Drug Administration (FDA) and the European Medicines Agency (EMA). These include the monoclonal antibodies (mAbs) trastuzumab [5] and pertuzumab [6], specifically targeting HER2. These also include the antibody drug conjugates trastuzumab emtansine, consisting of trastuzumab and a derivate of the cytotoxic tubulin polymerization inhibitor maytansine [7] and trastuzumab deruxtecan, an antibody-DNA topoisomerase 1 inhibitor conjugate [8]. Even though many patients respond well to these drugs, resistance develops in several cases [5], and a number of patients die of their HER2 overexpressing cancers.

This motivates development of HER2 specific drugs with other characteristics and modes of action. One possibility is to use a derivate of the well-studied *Pseudomonas aeruginosa* exotoxin A (ETA) where its natural targeting domain has been replaced with a targeting domain with specific affinity to the HER2 receptor. ETA is a highly cytotoxic enzyme that irreversibly modifies elongation factor 2 after entering the cell’s cytosol, and thereby shuts-down all protein synthesis, which leads to cell death. The most extensively studied ETA-derivate is PE38, where domain 1, which is responsible for its natural cell interaction, has been deleted. PE38-based cancer drugs appear to be generally safe in humans [9] and recently the first PE38-based drug, moxetumomab pasudotox, targeting cluster of differentiation-22 (CD22), was approved by the US Food and Drug Administration for clinical use on adults with relapsed or refractory hairy cell leukemia [10]. Engineering of PE38 by removal of potential mouse and human, B- and T-cell epitopes [11,12,13,14] has led to several deimmunized variants. One of them is PE38X8, with the amino acid alterations R313A, Q332S, R432G, R467A, R490A, R513A, R513A, E548S, K590S, to remove B-cell epitopes [12]. Another variant is PE25 where domain II was replaced with a furin cleavage site followed by the amino acids GGS as well as the deimmunizing amino acid alterations R313A, Q332S, R432G, R467A, R490A, R513A, R513A, E548S, K590S [15,16]. Both PE38X8 and PE25 are highly potent cytotoxic protein domains, which can act on a variety of cell lines, however, their in vivo characteristics are relatively unexplored.

The most frequently used targeting domains to direct ETA-derived toxins to tumor cells are antibody fragments. For example, moxetumomab pasudotox utilizes a variable fragment (Fv) with a Mw of approximately 25 kDa, derived from immunoglobulin G, to target CD22 [17].

In recent years, other types of targeting domains for ETA-derived toxins that are built on alternative, non-Ig scaffolds, have started to emerge. One class of such targeting domains are the affibody molecules. They are small (58 amino acids, Mw 7 kDa), robust protein domains that usually fold into an anti-parallel three helix bundle structure. Affibody molecules binding specifically to several cancer relevant cell surface receptors have been generated, including epidermal growth factor receptor (EGFR) [18], HER2 [19], human epidermal growth factor receptor 3 (HER3) [20], insulin-like growth factor 1 receptor (IGF1R) [21], and carbonic anhydrase IX (CAIX) [22]. Affibody molecules targeting different receptors appear to be generally safe when injected into humans [23]. The affibody molecule, Z_HER2:2891_, has strong affinity (equilibrium dissociation constant, K_D_, 66 pM) to HER2. It has previously been found to be able to specifically deliver PE38X8 to HER2 overexpressing cells [24]. Moreover, a ^68^Ga-labeled variant of Z_HER2:2891_, ^68^Ga-ABY-025, has shown excellent accumulation in metastases with HER2 expression in breast cancer patients, validating its ability for specific targeting of HER2 in a clinical context [25].

Another class of engineered scaffold proteins, developed as targeting domains, are the albumin binding domain-derived affinity proteins (ADAPTs). They consist of only 46 amino acids (Mw 5 kDa) and, like the affibody molecules, fold into an anti-parallel three helix bundle structure. ADAPTs with strong and specific affinity to different cancer relevant receptors have been developed, including HER2 and HER3 [26,27]. Studies with the HER2-binding radiolabeled ADAPT_6_ demonstrated that this engineered scaffold protein is capable of specific accumulation in HER2-expressing human xenografts in mice [28,29]. Moreover, clinical data show that radiolabeled ADAPT_6_ accumulates in HER2-expressing malignant breast tumors [30].

Fusion proteins consisting of affibody molecules or ADAPTs coupled to PE38X8 or PE25, result in constructs that will be smaller than the cut-off of the glomerular filtration in the kidneys, which is approximately 60 kDa, and will therefore likely be quickly cleared from circulation in vivo. ADAPT_6_-PE25, targeting HER2 overexpressing cells, was for example quickly cleared from circulation in mice; the concentration in blood was only 0.2 ± 0.1%ID/g at 4 h after injection [31]. Addition of an albumin binding domain (ABD) to proteins smaller than the cut-off of the glomerular filtration has previously been found to extend their plasma half-life [32]. When injected, such constructs immediately associate with albumin and the size of the complex is thereby increased by the molecular weight of albumin (67 kDa) to above the cut-off value. In addition, since albumin has an unusually long plasma half-life due to its interaction with the neonatal Fc receptor [33], fusion proteins including an ABD receive an extension of the plasma half-life from indirect interaction, via serum albumin, with the neonatal Fc receptor. The scaffold used for generation of ADAPTs is an albumin binding domain and the derivate ABD_035_ is an engineered version with femtomolar affinity (K_D_) for human serum albumin (HSA) [34].

Previously, we have found that ADAPT_6_ may be used for targeting of PE38X8 and PE25 to HER2 overexpressing cells [31]. However, biodistribution experiments in mice showed that the uptake varied considerably between different organs, a consequence of differences between PE38X8 and PE25. Significant differences were also found when comparing Z_HER2:2891_ and ADAPT_6_ as targeting domains for PE38X8. It is obvious that different constituents of a complex fusion protein may influence each other’s binding to their respective targets by e.g., imposing steric hindrance. Moreover, different constituents have different patterns of off-target interaction, which might enhance or attenuate uptake in normal tissues, and thus the biodistribution pattern of a fusion protein as well as its toxicity. This motivated us to extend our investigation of the properties of Z_HER2:2891_ as targeting domain of PE25 based fusion toxins.

In the present study, we created two affibody-PE25 fusion toxins. One utilizes Z_HER2:2891_ to target HER2 and the other utilizes a dual-HER2-binding domain consisting of Z_HER2:2891_ and ADAPT_6_. The fusion toxins were thoroughly characterized concerning biochemical properties and in vitro toxicity on cells with different expression level of HER2. The biodistribution of both constructs was also investigated head-to-head in mice.

## 2. Materials and Methods

### 2.1. General

All chemicals and reagents were from Sigma-Aldrich (Saint Louis, MO, USA) or Merck (Kenilworth, NJ, USA) unless otherwise noted. DNA restriction enzymes and ligase were from New England Biolabs (Ipswich, MA, USA).

### 2.2. Gene Construction

pET26-ADAPT_6_-ABD-PE25 [31] was used as starting point for construction of expression vectors. Its expression cassette consists of an N-terminal tag with the amino acid sequence HEHEHE followed by ADAPT_6_, an ABD and PE25. The vector was fitted with a *Nde*I and BamHI restriction sites surrounding ADAPT_6_. The expression vector for Z_HER2:2891_-ABD-PE25 was created by replacing ADAPT_6_ in pET26-ADAPT_6_-ABD-PE25 with Z_HER2:2891_, using the *Nde*I and *Bam*HI restriction sites. The expression vector for Z_HER2:2891_-ADAPT_6_-ABD-PE25 was created by inserting Z_HER2:2891_-ADAPT_6_, which was obtained by gene synthesis from Thermo Fisher Scientific (Waltham, MA, USA), using the *Nde*I and *Bam*HI restriction sites. The ABD used was ABD_035_, a version engineered for high affinity to human serum albumin [34]. PE25 is a deimmunized and minimized version of PE38 with the following amino acid alterations: R427A, F443A, D463A, R467A, L477H, R490A, R494A, R505A, R538A, L552E, and deletion of the majority of domain II (Δ251-273 and Δ285-394) [12]. A furin cleavage site is at the N-terminus of PE25 and is connected to domain III with a linker with the amino acid sequence GGS. All constructs were verified by DNA sequencing.

### 2.3. Protein Expression and Purification

*Escherichia coli* (BL21 Star (DE3)) (Merck Millipore, Billerica, Massachusetts, USA) was used as a host for protein expression. Overnight cultures of cells harboring the expression plasmids were prepared and diluted 1:100 in tryptic soy Broth (TSB) medium with yeast extract [31] and grown at 37 °C. When OD_600_ reached approximately 1.5, protein expression was induced by addition of Isopropyl β-D-1-thiogalactopyranoside to a final concentration of 1 mM. The cells were grown a further 2.5 h, after which they were harvested by centrifugation. The cell pellets were subsequently resuspended in TST-buffer (25 mM Tris(hydroxymethyl) aminomethane, 1 mM EDTA, 200 mM NaCl, 0.05% Tween 20 pH 8.0) supplemented with Complete EDTA-free protease inhibitor cocktail (Roche Diagnostic, Basel, Switzerland) and then broken by sonication. The fusion toxins were purified on an HiTrap NHS sepharose column (GE Healthcare Bio-Sciences, Uppsala, Sweden) with immobilized HSA, as previously described [31]. The proteins were eluted with 0.5 M acetic acid (pH 2.6) followed by buffer exchange to phosphate buffered saline (PBS). The molecular masses of the eluted proteins were determined by liquid chromatography electrospray ionization mass spectrometry on a Bruker impact II instrument (Agilent Technologies, Santa Clara, CA, USA), as previously described [31].

### 2.4. Biosensor Analysis

Biacore 3000 and T200 instruments (GE Healthcare, Uppsala, Sweden) were used for biosensor analysis of interaction with serum albumins and HER2, respectively. HER2 (the extracellular domain, Sino Biological, Beijing, China) was immobilized, by amine coupling, on a CM5 sensor chip (GE Healthcare) with sodium acetate buffer (pH 4.5) as immobilization buffer. HSA (Novozymes, Bagsvaerd, Denmark) and mouse serum albumin (MSA) (Sigma-Aldrich) were immobilized on a second CM5-chip. The final immobilization level of HER2 was 320 RU. HSA and MSA were immobilized to a final level of 265 and 207 RU, respectively. Reference flow cells were created by activation and deactivation on both chips. As running buffer and for dilution of the analytes, HBS-EP (10 mM HEPES, 150 mM NaCl, 3 mM EDTA, 0.05% Tween 20, pH 7.4) was used. All experiments were performed at 25 °C with a flow rate of 50 μL/min.

### 2.5. Cell Culture 

SKOV3, SKBR-3, AU565, and A549 cell lines were obtained from the American Type Culture Collection (ATCC) and were grown in McCoy’s 5a or Dulbecco’s Modified Eagle’s medium in a humidified incubator at 37 °C in 5% CO_2_ atmosphere. The cells were grown for a maximum of 3 months after which the cell cultures were restarted from a frozen aliquot from the cell bank.

### 2.6. Flow Cytometry Analysis

To evaluate binding to HER2 on cells, SKOV3 (with high HER2 expression) was used. Samples of 1 × 10^5^ cells were collected and incubated with different concentrations of the fusion toxins, diluted in 100 μL PBSB-buffer (PBS with 1% BSA) for 2 h at 25 °C. Then, the cells were counterstained with Alexa Fluor 647-labeled HSA. Between each incubation step, the cells were washed once with PBSB. The fluorescence of the cells was measured in a Galios flow cytometer (Beckman Coulter, Stockholm, Sweden), where 10,000 events were recorded for each sample.

### 2.7. Radiolabeling with ^99m^Tc and ^111^In

Labeling of Z_HER2:2891_-ABD-PE25 and Z_HER2:2891_-ADAPT_6_-ABD-PE25 with ^99m^Tc for measurement of their binding to living cells was performed according to methodology described earlier [35].

Briefly, a ^99^Mo/^99m^Tc generator eluate containing ^99m^Tc-TcO_4_^-^ (500 µL, 3–3.5 GBq) was added to a vial containing CRS kit (PSI, Villigen, Switzerland). The sealed vial was incubated at 100 °C for 30 min. Thereafter, 15 µL of solution containing ^99m^Tc(CO)_3_ was transferred to a vial containing 75 µg of protein in 85 µL PBS. After 60 min incubation at 42 °C, a 5000-fold excess of histidine was added, and the mixture was incubated for 30 min at 42 °C. Radiolabeled proteins were purified by passage through a NAP-5 size-exclusion column (GE Healthcare). The radiochemical purities of the labelled proteins were 98.8–99%.

Labeling of Z_HER2:2891_-ABD-PE25 and Z_HER2:2891_-ADAPT_6_-ABD-PE25 with ^111^In for biodistribution measurements was performed as previously described for ADAPT_6_-ABD-PE25 [31]. Briefly, a CHX-A’’-DTPA chelator was conjugated to the fusion toxins by incubation of 1.1-fold molar excess of the chelator with protein in 0.07 M sodium borate, pH 9.3, at room temperature overnight. Thereafter, an excess of unreacted chelator was removed, and buffer was changed by passing the mixture through a NAP-5 column, pre-equilibrated and eluted with 0.2 M ammonium acetate (pH 5.5). For labeling, 200 µg conjugate was mixed with ^111^InCl (8–10 MBq in 0.05 M HCl), and the mixture was incubated for 60 min at room termperature. The radiolabeled proteins were purified using NAP-5 size-exclusion columns. The radiochemical purities of conjugates were over 95%.

### 2.8. Binding Studies Using a Ligandtracer Instrument

Affinity measurements against HER2 expressing cells were performed in real time using a Ligandtracer instrument (Ridgeview Diagnostics, Uppsala, Sweden) [36]. For this, SKOV3 cells (2 × 10^6^) were seeded in one area of a 10 cm petri dish one day before the experiment. After adding 3 mL of complete media, the dish was placed on the rotating table of the instrument and measured for 30 min as baseline. Two concentrations of each ^99m^Tc labeled fusion toxin (6 and 18 nM) were subsequently added to the cells and each concentration was measured for 90 min. For the dissociation phase, the medium was replaced with 3 mL of fresh medium and the measurement was continued overnight. The experiment was performed in duplicate at room temperature.

To evaluate the kinetics and stoichiometry of the interaction, an Interaction Map (Ridgeview Diagnostics) analysis was performed for each curve and the results were plotted by Tracedrawer software (Ridgeview Diagnostics).

### 2.9. Cell Binding Specificity Test

Approximately 1 × 10^6^ SKOV3 cells/well were seeded in a 6-well plate. On the following morning cells were washed once with 1 mL complete medium. Then, 1000 nM of non-labeled compound in 500 µL medium, or same volume of fresh medium, was added to each well (*n* = 3) 15–30 min prior to addition of radiolabeled compound. Next, 4 nM of radiolabeled fusion toxin was added to each well to a final concentration of 2 nM and the 6-well plate was incubated at 37 °C for 1 h. After incubation, the wells were washed twice with PBS and the cells were detached by addition of a Trypsin-EDTA solution. The radioactivity of the cells was analyzed in an automated gamma-counter.

### 2.10. In Vitro Cytotoxicity Analysis

Cell viability was assessed by measurement of intracellular dehydrogenases activity using the Cell counting kit-8 assay (Sigma-Aldrich). To determine the cytotoxicity of each fusion toxin for each cell line, approximately 5000 cells/well were seeded in a 96-well plate and were allowed to attach for 4 h. The medium was then replaced with fresh medium containing the indicated concentrations of the fusion toxins, followed by incubation for 72 h at 37 °C. The cells were washed with PBS, and viability was measured according to the supplier’s protocol, with determination of A_450_ in each well. The obtained absorbance values were plotted and analyzed by Prism 8 (GraphPad Software, La Jolla, CA, USA).

### 2.11. Biodistribution Experiments

The animal experiments were planned and performed according to Sweden’s national legislation on laboratory animals’ protection. The animal studies were approved (project identifier: 86/15) by the local ethics committee for animal research in Uppsala, Sweden, on 18 June 2016.

Biodistribution of ^111^In-labeled Z_HER2:2891_-ABD-PE25 and Z_HER2:2891_-ADAPT_6_-ABD-PE25 was measured in exactly the same way as biodistribution of ^111^In-ADAPT_6_-ABD-PE25 previously [31], using female NMRI mice (Taconic Biosciences, Ejby, Denmark). The mice were 10 weeks old upon arrival, and their weight was 26 ± 2 g at the time of the experiment. A group of four mice was used per data point. Euthanasia was performed using i.p. injection of a mixture of Ketalar (200 mg/kg body weight) and Rompun (20 mg/kg body weight).

^111^In-Z_HER2:2891_-ABD-PE25 and ^111^In-Z_HER2:2891_-ADAPT_6_-ABD-PE25 (1 µg/20 kBq in 100 μL PBS containing 2% BSA per animal) were injected in the tail veins of the mice. At 4 and 24 h after injection, the animals were injected with a lethal dose of anesthetic and exsanguinated using a heparinized syringe. Organs and tissue samples were collected, weighed, and their radioactivity was measured using an automated gamma-spectrometer.

## 3. Results

### 3.1. Construct Design, Protein Expression, and Purification

**Figure 1:**
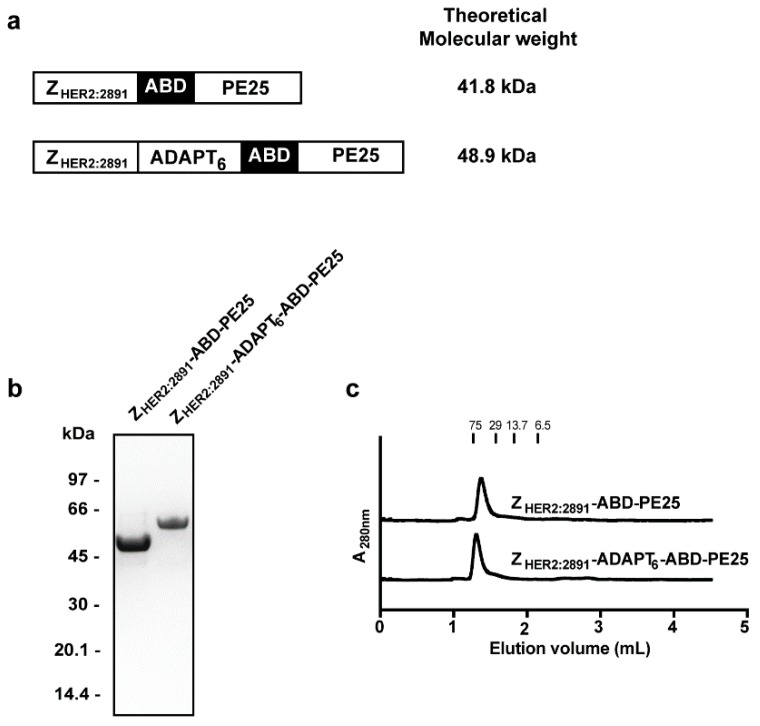
Schematic description and initial biochemical characterization of the fusion toxins. (**a**) A schematic description of the fusion toxins analyzed in the current study along with their theoretical molecular weights. The individual domains of the fusion toxins were connected with linkers with the amino acid sequence (S_4_G)_4_. After purification by affinity chromatography with immobilized human serum albumin (HSA), the fusion toxins were analyzed by sodium dodecyl sulfate–polyacrylamide gel electrophoresis (SDS-PAGE) under reducing conditions (**b**). The numbers to the left indicate molecular weights of reference proteins. The fusion toxins were also analyzed by analytical size exclusion chromatography and the recorded chromatograms are shown in (**c**). The numbers above the chromatograms correspond to elution volumes of reference proteins.

The HER2 specific fusion toxins are schematically outlined in Figure 1a. The targeting part consists of the HER2 specific affibody molecule Z_HER2:2891_ or the dual HER2 targeting hybrid, Z_HER2:2891_-ADAPT_6_. An albumin binding domain (ABD) was included in both constructs for in vivo half-life extension. In both cases the domain PE25, a deimmunized and truncated version of *Pseudomonas aeruginosa* exotoxin A, was used for its cytotoxic capacity. All domains were connected with linkers with the amino acid sequence (S_4_G)_4_ and both fusion toxins were fitted with an N-terminal tag with the amino acid sequence HEHEHE, suitable for radionuclide chelation. The fusion toxins were expressed in the cytoplasm of *Escherichia coli*. The strong HSA affinity presented by ABD was capitalized upon, and the fusion toxins were purified by a single affinity chromatographic step, utilizing a column with immobilized HSA. Eluted material was pooled and analyzed by sodium dodecyl sulfate–polyacrylamide gel electrophoresis (SDS-PAGE) showing pure and homogenous proteins (Figure 1b). The molecular weights of the proteins were confirmed by mass spectrometry (Figure S1) to be 41.8 and 48.9 kDa for Z_HER2:2891_-ABD-PE25 and Z_HER2:2891_-ADAPT_6_-ABD-PE25, respectively. The proteins were further analyzed by passage through a size-exclusion chromatography column and were eluted as single symmetrical peaks with retention times expected of monomers, showing that no di- or multimers were formed (Figure 1c).

### 3.2. Interaction of the Fusion Toxins with HER2

**Figure 2:**
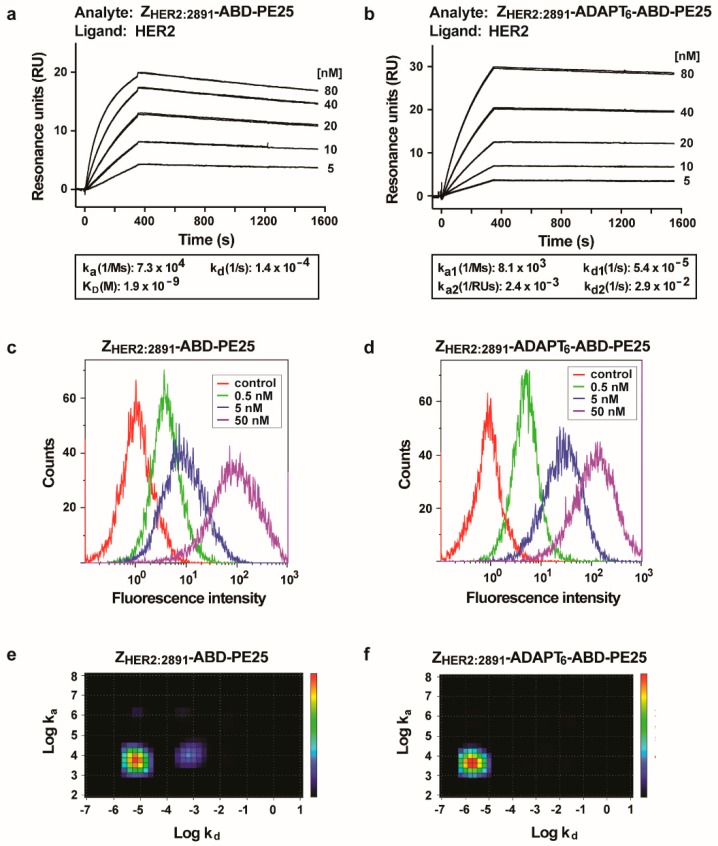
Analysis of the interaction with HER2. (**a**,**b**) Sensorgrams after analyses using a Biacore real-time biosensor of the interaction of the fusion toxins with HER2. Dilution series of the fusion toxins were injected sequentially over a surface with immobilized HER2. Each concentration was injected twice, and each panel is an overlay of both repeats of all concentrations. The injected concentrations are indicated to the right of each panel. SKOV3 cells were stained with different concentrations of the fusion toxins, followed by staining with fluorescently labeled human serum albumin (HSA) and were analyzed by flow cytometry (**c**,**d**). The concentration of the fusion toxins used for staining is indicated in the legend. Each panel is an overlay of the plots obtained for all concentrations for each fusion toxin. The controls are non-stained cells. Further, the fusion toxins were radiolabeled with ^99m^Tc and incubated with SKOV3 cells and cell bound radioactivity was recorded as a function of time in a Ligandtracer instrument. The obtained curves were analyzed by Tracedrawer software and the resulting Interaction Maps are presented in (**e**,**f**).

The interaction of the fusion toxins with HER2 was investigated by real time biosensor analyses on a Biacore instrument. HER2 was immobilized on the chip surface and dilution series of the fusion toxins were sequentially injected (Figure 2a,b). A concentration dependent response was recorded after blank subtraction, showing that both fusion toxins could interact with HER2. For Z_HER2:2891_-ABD-PE25, the kinetic constants were derived assuming a 1:1 interaction and K_D_ was determined to 1.9 nM. Since Z_HER2:2891_-ADAPT_6_-ABD-PE25 contains two HER2-interacting domains, the kinetic constants for interaction with HER2 cannot be derived assuming a 1:1 interaction model. In this case the kinetic constants were derived assuming a bivalent analyte binding model. Visual comparison of the dissociation phase in Figure 2a,b shows that the off-rate is slower for Z_HER2:2891_-ADAPT_6_-ABD-PE25 compared to Z_HER2:2891_-ABD-PE25, suggesting an avidity effect of the two binding domains.

Further, the interaction of the fusion toxins with cells were investigated by flow cytometry. SKOV3 cells with high HER2 expression were incubated with the fusion toxins followed by detection with fluorescently labeled HSA that should bind to the ABD-part. A concentration dependent response was obtained showing that the fusion proteins could indeed interact with SKOV3 cells (Figure 2c,d).

To determine the K_D_ of the interactions between the radiolabeled fusion toxins and SKOV3 cells, the HEHEHE-tag of the fusion toxins were radiolabeled with ^99m^Tc and added to a rotating dish with SKOV3 cells in a Ligandtracer instrument. The results were analyzed by Interaction Map software to determine the K_D_ and stoichiometry of the interactions (Figure 2e,f). For both fusion toxins, the interaction was dominated by a primary event with a minor secondary event, showing that in both cases the stoichiometry of the interaction is primarily a 1:1 interaction. Surprisingly, the secondary event is more pronounced for the Z_HER2:2891_-ABD-PE25/SKOV3 interaction, that theoretically is a 1:1 interaction, than for the Z_HER2:2891_-ADAPT_6_-ABD-PE25/SKOV3 interaction, that theoretically is a 2:1 interaction. The secondary event for the Z_HER2:2891_-ADAPT_6_-ABD-PE25/SKOV3 interaction might be difficult to see in Figure 2f, and accounts for <1% of the interaction. The primary event for the interaction between Z_HER2:2891_-ABD-PE25 and SKOV3 is centered around a K_D_ of 1.8 nM. The primary event for the Z_HER2:2891_-ADAPT_6_-ABD-PE25/SKOV3 interaction is centered around a K_D_ of 0.47 nM. The results showed that the interaction between both fusion toxins and SKOV3 is similar. It also suggests that one of the HER2 binding domains of Z_HER2:2891_-ADAPT_6_-ABD-PE25 is contributing almost exclusively to the interaction with the cells.

To ascertain that the radiolabeled fusion toxins did not stick non-specifically to the SKOV3 cells, a binding specificity experiments was performed (Figure S2). In that experiment, cells were incubated with a radiolabeled fusion toxin with or without pre-incubation with an excess of non-labeled fusion toxin. The results showed that for both fusion toxins, the radioactive signal could to a large extent be blocked by addition of an excess of cold fusion toxin, suggesting that the interaction of the radiolabeled fusion toxin with the cells was through the HER2 receptor.

### 3.3. Analysis of Affinity to Serum Albumins

**Figure 3:**
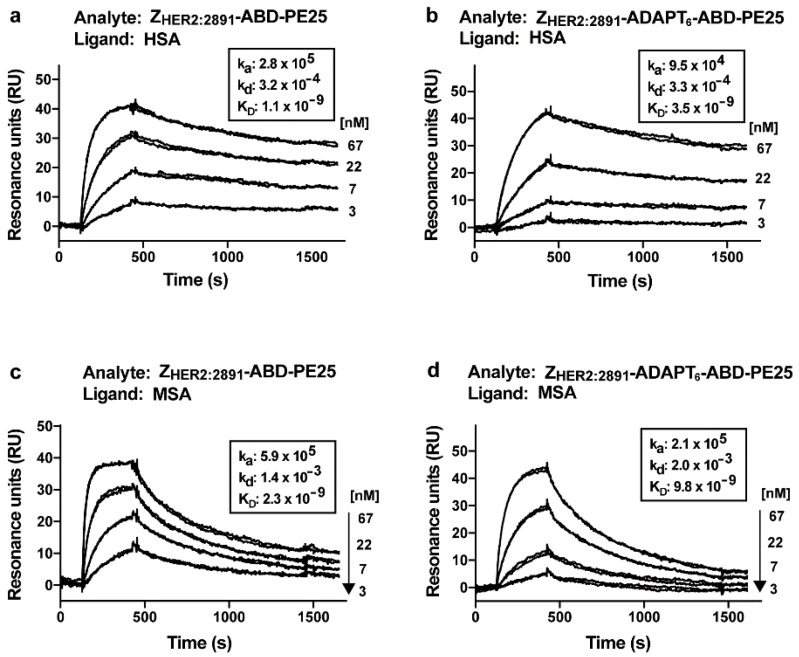
Analysis of the interaction between the fusion toxins and HSA and mouse serum albumin (MSA). The analyses were carried out a Biacore real-time biosensor instrument. A dilution series of Z_HER2:2891_-ABD-PE25 was injected over a surfaces with immobilized HSA (**a**); a dilution series of Z_HER2:2891_-ADAPT_6_-ABD-PE25 was injected over a surface with immobilized HSA (**b**); a dilution series of Z_HER2:2891_-ABD-PE25 was injected over a surfaces with immobilized MSA (**c**); a dilution series of Z_HER2:2891_-ADAPT_6_-ABD-PE25 was injected over a surface with immobilized MSA (**d**). The concentrations are indicated to the right of each panel. Each concentration was injected twice and each panel is an overlay of the sensorgrams recorded for both repeats of each concentration for the whole dilution series.

The affinities of the fusion toxins to HSA and mouse serum albumin (MSA) were determined by real-time biosensor analyses on a Biacore instrument. To determine the kinetic constants and equilibrium dissociation constant (K_D_) of the interactions, dilution series of Z_HER2:2891_-ABD-PE25 and Z_HER2:2891_-ADAPT_6_-ABD-PE25 were injected over surfaces with immobilized HSA or MSA (Figure 3). The on-rate for Z_HER2:2891_-ADAPT_6_-ABD-PE25 was slower than for Z_HER2:2891_-ABD-PE25 on both the HSA and MSA surfaces. The off-rate of both fusion toxins on both surfaces were similar. This resulted in a slightly stronger affinity of Z_HER2:2891_-ABD-PE25 compared to Z_HER2:2891_-ADAPT_6_-ABD-PE25 on both the HSA and MSA surfaces. The K_D_ was 1.1 and 3.5 nM for the interaction between Z_HER2:2891_-ABD-PE25 or Z_HER2:2891_-ADAPT_6_-ABD-PE25 and the HSA surface, respectively. The interaction with the MSA surface was weaker, 2.3 and 9.8 nM, respectively, for Z_HER2:2891_-ABD-PE25 or Z_HER2:2891_-ADAPT_6_-ABD-PE25.

### 3.4. Cytotoxicity Analysis

**Figure 4:**
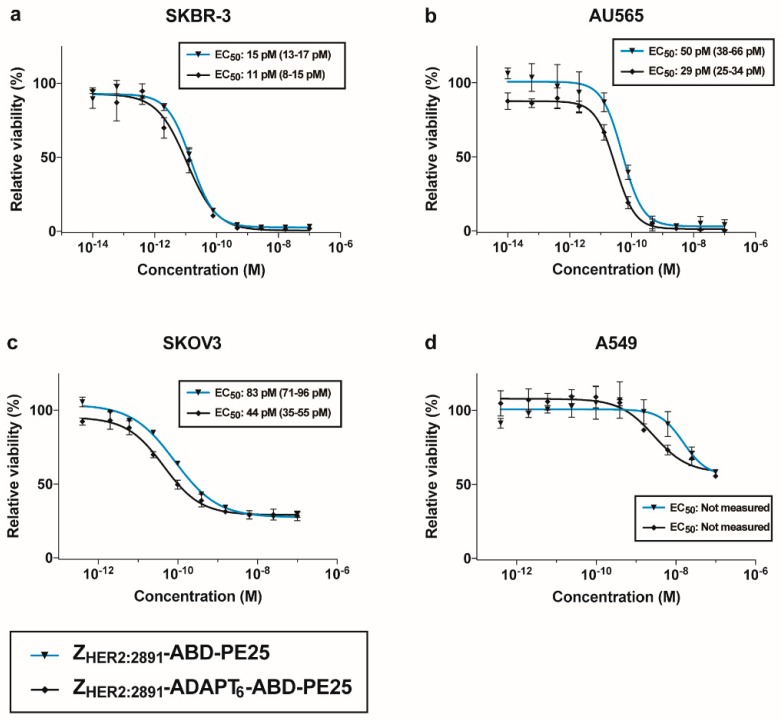
Determination of cytotoxic potency of fusion toxins. SKBR-3 (**a**), AU565 (**b**), SKOV3 (**c**) and A549 (**d**) cells were incubated with different concentrations of the fusion toxins and the viability of the cells was measured as a function of protein concentration. The viability of cells incubated without toxin (control cells) was set to 100%. The measured viability was plotted as percent viability compared to control cells on the y-axis, as a function of the concentration of fusion toxin on the x-axis. Each data-point corresponds to the average viability of four independent experiments. The error bars correspond to 1 SD.

The cytotoxicity was determined by incubating dilution series of the fusion toxins with different cell lines (Figure 4). For high HER2 expressing cells: AU565 (breast carcinoma), SKBR-3 (breast carcinoma), and SKOV3 (ovarian carcinoma) the EC_50_ was between 10^−10^ and 10^−11^ M. This shows that the toxins are extremely potent on cells with high HER2 expression derived from epithelial tissues of different origin. Both fusion toxins were considerably less toxic to A549 (lung carcinoma), derived from epithelial tissue and with medium HER2 expression.

### 3.5. Biodistribution

The two fusion toxins were radiolabeled with ^111^In and injected into mice and data concerning biodistribution of the fusion toxins were collected and are presented in Table 1. For comparison, historical data concerning the biodistribution of ADAPT_6_-ABD-PE25 are included in the table [31]. Measurements performed 4 h after injection showed that blood retention was significantly higher for Z_HER2:2891_-ABD-PE25 compared to the other two. At 24 h the blood retention was significantly higher for Z_HER2:2891_-ABD-PE25 compared to Z_HER2:2891_-ADAPT_6_-ABD-PE25. These results show that Z_HER2:2891_-ABD-PE25 has the longest plasma half-life. Liver uptake was significantly lower for Z_HER2:2891_-ABD-PE25 compared to ADAPT_6_-ABD-PE25. Conversely, kidney uptake at 4 h was lower for ADAPT_6_-ABD-PE25 compared to Z_HER2:2891_-ABD-PE25. Uptake in liver and kidneys of Z_HER2:2891_-ADAPT_6_-ABD-PE25, containing the dual-targeting domain was between the values measured for Z_HER2:2891_-ABD-PE25 and ADAPT_6_-ABD-PE25. The same pattern was seen at 24 h, except that Z_HER2:2891_-ADAPT_6_-ABD-PE25 had a higher uptake in liver compared to the other two. Collectively these results suggest that Z_HER2:2891_ promotes a clearance route through the kidneys and that ADAPT_6_ promotes a clearance route through the liver for PE25-based fusion toxins.

Uptake in the spleen was also significantly lower for both fusion toxins containing Z_HER2:2891_ compared to ADAPT_6_-ABD-PE25. This suggests that ADAPT_6_ promotes uptake in spleen, which has also been seen earlier [28]. The promoted uptake in spleen by ADAPT_6_ can be counteracted by Z_HER2:2891_.

## 4. Discussion

In the present study, we investigated different targeting domains for delivery of PE25-based fusion toxins to HER2 overexpressing cells. We designed two toxin fusions, Z_HER2:2891_-ABD-PE25 and Z_HER2:2891_-ADAPT_6_-ABD-PE25, and investigated their properties in vitro and in vivo.

Both fusion toxins could be produced in a soluble form in *Escherichia coli* and efficiently purified to homogeneity by a single affinity chromatography step. This is an advantage from a manufacturing point of view compared to immunotoxins utilizing e.g., an immunoglobulin-derived Fv fragment. Although such immunotoxins can also be expressed in *Escherichia coli*, they typically form inclusion bodies [37] that require solubilization and refolding, which leads to a much more cumbersome purification process.

Interaction analyses in vitro were performed by several different methods. Determination of the kinetic constants by real-time biosensor interaction in a Biacore instrument for Z_HER2:2891_-ABD-PE25 for HER2 is straight-forward as it should follow a Langmuir 1:1 interaction. To analyze the interaction between Z_HER2:2891_-ADAPT_6_-ABD-PE25 and HER2 is more difficult, since it contains two different targeting domains. A possibility is to utilize the bivalent analyte binding model in the Biacore software package, as we have done in this study. However, it assumes that the two HER2-binding parts of Z_HER2:2891_-ADAPT_6_-ABD-PE25 are interacting identically with HER2, as would e.g., the two antigen binding arms of an antibody. Since the kinetic constants for the interaction between ADAPT_6_-ABD-PE25 and HER2 [31] is different from the kinetic constants derived for the interaction between Z_HER2:2891_-ABD-PE25 and HER2 in the present study, the kinetic constants derived from the bivalent analyte binding model for Z_HER2:2891_-ADAPT_6_-ABD-PE25 can only be regarded as an approximation. A possibility would be to reverse the set-up and immobilize the fusion toxins on the chip-surface followed by injection of soluble HER2. However, that experimental set-up is inferior to the current one in terms of modeling binding to cells, since HER2′s natural context is on the cell surface. Binding to cells was instead investigated by incubating SKOV3 cells with different concentration of the fusion toxins, followed by staining with fluorescently labeled HSA and analysis by flow cytometry, showing an increase in fluorescence signal with increasing concentration. An important observation from this experiment is that both fusion toxins could bind to HSA and HER2 simultaneously. Due to the ubiquitously present HSA in the blood stream, simultaneous binding to HER2 and HSA will likely be required for efficient tumor targeting in vivo. The kinetic constants and the affinity between the fusion toxins and SKOV3 cells were determined using a Ligandtracer instrument followed by analysis by Tracedrawer software. Interestingly, the primary event in the interaction between Z_HER2:2891_-ABD-PE25 and HER2 was 1.8 nM, which was close to the K_D_-value for the same interaction determined in the Biacore instrument (1.9 nM). This shows that the set-up in the Biacore mimics the interaction with the cells relatively well in this case, despite that HER2 is non-specifically attached to a dextran coated surface and not properly embedded in a cell membrane. However, it is apparent that the affinity of both fusion toxins for HER2 is weaker than the affinity of the individual domains for HER2. The K_D_-values of Z_HER2:2891_ and ADAPT_6_ for interaction with HER2 have previously been determined to 66 pM and 0.5 nM, respectively [26,38]. A weaker affinity to HER2, when fused to truncated derivatives of ETA, has also been recorded previously for Z_HER2:2891_ and ADAPT_6_ [24,31]. An unexpected result of the Ligandtracer affinity measurement was that the interaction between Z_HER2:2891_-ADAPT_6_-ABD-PE25 and HER2 was dominated by one single event and not two. However, the off-rate of Z_HER2:2891_-ADAPT_6_-ABD-PE25 in the Biacore analysis was slower than the off-rate of Z_HER2:2891_-ABD-PE25, indicating a cooperativity in binding of both domains. An alternative explanation is that flanking ADAPT_6_ with two domains (Z_HER2:2891_ and ABD-PE25) prevents its binding to HER2 on living cells due to steric hindrance. In this scenario however, ADAPT_6_ might act as an additional spacer between Z_HER2:2891_ and PE25, permitting more efficient binding of the affibody molecule to HER2.

The affinity of the fusion toxins to HSA and MSA was also determined in a Biacore instrument, and was similar to the interaction of other fusion proteins containing the same ABD-domain [31]. Consistent with observations on fusion toxins containing the same ABD, the affinity of both fusion toxins was stronger to HSA than to MSA [31].

A relatively long linker of 20 amino acids, dominated by serine, was used to connect all domains in both fusion toxins. The rationale behind this choice was that since both HER2 and HSA are relatively large compared to Z_HER2:2891_, ADAPT_6_, and ABD, long linkers could be necessary to allow simultaneous binding. Even though we did not compare different linkers in this study, is was clearly demonstrated by the flow cytometry experiments (Figure 2c,d), that the length was enough to allow simultaneous binding of HER2 and HSA. Investigation of different linkers could be an interesting follow-up study to the present one, and linker length could perhaps affect triggering of HER2 signaling or rate of internalization. Previous studies have shown that dimeric HER2 binding affibody molecules can affect signaling through e.g., the mitogen-activated protein kinase MAPK pathway, although the effect on e.g., cell proliferation was different for different cell lines [39,40]. Additionally, dimeric HER2 binding designed ankyrin repeat proteins, with different linker lengths, gave rise to different cytotoxic responses in HER2 overexpressing cell lines [41]. A difference between the two studies on dimeric affibody constructs and the study on dimeric designed ankyrin repeat proteins, was that a homo-dimer was used in the affibody studies but the designed ankyrin repeat proteins consisted of two domains binding to different parts of HER2, which is more similar to the dual-targeting domain, Z_HER2:2891_-ADAPT_6_, used in this study.

The toxicity (EC_50_-value) of both fusion toxins to SKBR-3, SKOV3, and AU565 cells, all with high HER2 expression, was between 10 and 100 pM. This is similar to the EC_50_-values found previously for ADAPT_6_-ABD-PE25 on the same cell lines [31]. Since the affinity of ADAPT_6_-ABD-PE25 for HER2 is 10-fold weaker (15 nM) [31] compared to the affinity between Z_HER2:2891_-ABD-PE25 and HER2 (1.9 nM), it appears that an affinity stronger than “low nanomolar” does not increase cytotoxicity of PE25-based fusion toxins towards these cell lines. However, it should be noted that the comparison between ADAPT_6_ and Z_HER2:2891_ is an approximation, since they might affect internalization of HER2 differently. An additional result that points towards that ADAPT_6_ and Z_HER2:2891_ confers a similar cytotoxicity in PE25-based constructs is that Z_HER2:2891_-ADAPT_6_-ABD-PE25 shows similar cytotoxicity towards the HER2 overexpressing cell lines. This fusion toxin has an even stronger affinity to HER2 on SKOV3 cells than ADAPT_6_-ABD-PE25 and Z_HER2:2891_-ABD-PE25.

The in vivo biodistribution experiment suggested that the clearance route of the fusion toxins, when utilizing Z_HER2:2891_ as targeting domain, was mainly through the kidneys, in contrast to the use of ADAPT_6_ as targeting domain of PE25 which had a more pronounced clearance through the liver [31]. In Table 1, the uptake of Z_HER2:2891_-ABD-PE25 in the GI-tract at 24 h is also significantly lower than the other two, as a consequence of the shift in clearance route to the kidneys. Clearance through the kidneys is more desirable than liver, since liver damage is a more common side effect of targeted cytotoxic drugs such as antibody drug conjugates [42]. It should be noted that neither Z_HER2:2891_ nor ADAPT_6_ is cross-reactive with murine HER2, so the uptake in different organs was not mediated by interaction with HER2. This means that the observed differences for the fusion toxins is not obscured by endogenous murine HER2 receptors in the mice, that could have contributed to a difference in the uptake of Z_HER2:2891_ or ADAPT_6_.

A possible continuation of the current study could be to investigate the efficacy of the fusion toxins in animal models with implanted HER2 expressing tumors. Of particular interest would be to determine if tumor growth can be slowed and survival of the animals can be prolonged, similar to what has previously been demonstrated for other affibody based fusion toxins [43,44]. Of particular interest would be to compare liver uptake and maximum tolerated dose for different variants, to get a general understanding of whether it is liver toxicity or something else that determines the maximum tolerated dose of *Pseudomonas* Exotoxin A-based fusion toxins.

Cancer treatment regimens for patients with HER2 overexpression routinely includes HER2 specific antibodies or antibody-drug conjugates. Resistance to the drugs develops in some patients, but the tumors may still overexpress HER2 [45], which would allow treatment with potential drugs such as the fusion toxins described in this study. Additionally, since Z_HER2:2891_ interacts with domain III of HER2 [46], at a site which is distinct from the site of interaction with trastuzumab and pertuzumab, a combination therapy of the clinically validated antibodies or antibody drug conjugates and the Z_HER2:2891_-containing fusion toxins should be possible, which could lead to higher efficacy than either compound alone.

In conclusion, Z_HER2:2891_ and Z_HER2:2891_-ADAPT_6_ are efficient targeting domains for PE25-based fusion toxins, resulting in agents with high cytotoxicity and specificity for HER2 overexpressing cells. The cytotoxicity profiles and distribution in vivo suggest that Z_HER2:2891_ is superior compared to Z_HER2:2891_-ADAPT_6_ and ADAPT_6_ as a targeting domain in the context of the present study.

## Figures and Tables

**Table 1:** Comparative biodistribution of the ^111^In-labeled fusion toxins in mice 4 and 24 h following intravenous injection ^a^.

	Z_HER2:2891_-ABD-PE25	ADAPT_6_-ABD-PE25 ^e^	Z_HER2:2891_-ADAPT_6_-ABD-PE25
	At 4 h post-injection		
Blood	9.2 ± 1.0 ^c,d^	3.2 ± 0.1	4.6 ± 0.9
Heart	3.0 ± 0.5 ^c,d^	1.1 ± 0.2	1.7 ± 0.2
Lung	4.0 ± 0.5 ^c,d^	1.3 ± 0.1 ^d^	2.2 ± 0.5
Salivary gland	1.6 ± 0.4 ^c,d^	0.7 ± 0.2	0.8 ± 0.3
Liver	13.9 ± 2.2 ^c^	42.4 ± 6.8 ^d^	23.1 ± 3.8
Spleen	6.2 ± 2.0 ^c^	18.9 ± 4.2 ^d^	7.8 ± 2.0
Pancreas	0.9 ± 0.1	0.4 ± 0.1	1.0 ± 0.5
Stomach	1.0 ± 0.1 ^c^	0.5 ± 0.2	0.7 ± 0.1
Kidney	40.1 ± 4.8 ^c,d^	12.5 ± 0.6 ^d^	69.7 ± 8.0
Colon	1.3 ± 0.1 ^c^	0.6 ± 0.3	1.0 ± 0.3
Skin	1.5 ± 0.5 ^c^	0.6 ± 0.1	0.9 ± 0.2
Muscle	0.7 ± 0.1 ^c,d^	0.3 ± 0.1	0.4 ± 0.1
Bone	1.4 ± 0.1 ^d^	1.1 ± 0.3	0.9 ± 0.2
GI tract ^b^	3.5 ± 0.5	6.9 ± 4.4	3.3 ± 1.1
Carcass ^b^	17.8 ± 2.1 ^c,d^	3.5 ± 2.8 ^d^	13.0 ± 0.8
	At 24 h post-injection		
Blood	2.6 ± 0.5 ^d^	2.0 ± 0.1	1.8 ± 0.3
Heart	1.6 ± 0.3	1.8 ± 0.2 ^d^	1.2 ± 0.1
Lung	1.8 ± 0.3 ^d^	1.7 ± 0.2 ^d^	1.2 ± 0.1
Sal gland	1.2 ± 0.3 ^d^	1.2 ± 0.1 ^d^	0.8 ± 0.1
Liver	8.7 ± 0.9 ^c,d^	21.9 ± 2.0	16.2 ± 1.7
Spleen	5.5 ± 1.1 ^c^	8.5 ± 1.7 ^d^	5.3 ± 1.3
Pancreas	0.6 ± 0.1	0.6 ± 0.1	0.5 ± 0.1
Stomach	0.8 ± 0.1	0.7 ± 0.1	0.6 ± 0.1
Kidney	33.4 ± 4.4 ^c,d^	9.3 ± 1.3 ^d^	47.1 ± 5.2
Colon	0.5 ± 0.1 ^c^	0.9 ± 0.1 ^d^	0.5 ± 0.1
Skin	2.0 ± 0.4 ^c,d^	1.2 ± 0.1	1.3 ± 0.1
Muscle	0.5 ± 0.1	0.5 ± 0.1	0.4 ± 0.0
Bone	1.6 ± 0.5	1.8 ± 0.3	0.9 ± 0.2
GI tract ^b^	1.5 ± 0.1 ^c^	2.7 ± 0.7 ^d^	1.0 ± 0.1
Carcass ^b^	16.4 ± 1.5 ^c,d^	13.3 ± 1.4	11.8 ± 1.1

^a^ The measured radioactivity of different organs is expressed as %ID/g and presented as an average value from four animals ± 1 SD. ^b^ Data for gastrointestinal (GI) tract with content and carcass are presented as %ID per whole sample. Data were assessed by one-way ANOVA with Bonferroni’s post hoc multiple comparisons test in order to determine significant differences between groups (*p* < 0.05) at the same time point. No statistical analysis of values obtained for the same construct in the same organ at the two time-points was performed. ^c^ *p* < 0.05 vs. ADAPT_6_-ABD-PE25. ^d^ *p* < 0.05 vs. Z_HER2:2891_-ADAPT_6_-ABD-PE25. ^e^ Historical data [31].

## Supplementary Materials

The following are available online at https://www.mdpi.com/1999-4923/12/4/391/s1; Figure S1: Analysis of molecular masses by mass spectrometry; Figure S2: Specificity of binding.
