# Supplementary material for: HER2-Specific Pseudomonas Exotoxin A PE25 Based Fusions: Influence of Targeting Domain on Target Binding, Toxicity, and In Vivo Biodistribution
Source: Pharmaceutics. 2020 Apr 24;12(4):391. doi: 10.3390/pharmaceutics12040391 (PMC7238247; doi:10.3390/pharmaceutics12040391)
Supplement: Supplementary file 1 [file pharmaceutics-12-00391-s001.pdf]

pharmaceutics

Supplementary Materials

HER2-Specific Pseudomonas Exotoxin A PE25 Based Fusions: Influence of Targeting Domain on Target Binding, Toxicity, and In Vivo Biodistribution

Haozhong Ding [1,†], Mohamed Altai [2,†], Wen Yin [1], Sarah Lindbo [1], Hao Liu [1], Javad Garousi [3], Tianqi Xu [3], Anna Orlova [4,5], Vladimir Tolmachev [3,5], Sophia Hober [1] and Torbjörn Gräslund [1,*]

1. Department of Protein Science, KTH Royal Institute of Technology, Roslagstullsbacken 21, 114 17 Stockholm, Sweden; haozhong@kth.se (H.D.); wenyin@kth.se (W.Y.); slindbo@kth.se (S.L.); haoliu2@kth.se (H.L.); sophia@kth.se (S.H.)
2. Department of Oncology and Pathology, Barngatan 4, Lund University, 222 42 Lund, Sweden; mohamed.altai@med.lu.se
3. Department of Immunology, Genetics and Pathology, Dag Hammarskjölds väg 20, Uppsala University, 751 85 Uppsala, Sweden; javad.garousi@igp.uu.se (J.G.); tianqi.xu@igp.uu.se (T.X.); vladimir.tolmachev@igp.uu.se (V.T.)
4. Department of Medicinal Chemistry, Dag Hammarskjölds väg 14C; Uppsala University, 751 23 Uppsala, Sweden; anna.orlova@ilk.uu.se
5. Research Centrum for Oncotheranostics, Research School of Chemistry and Applied Biomedical Sciences, Tomsk Polytechnic University, 634050 Tomsk, Russia.

* Correspondence: torbjorn@kth.se; Tel.: +46-(0)8-790-9627

† These authors contributed equally to this work.

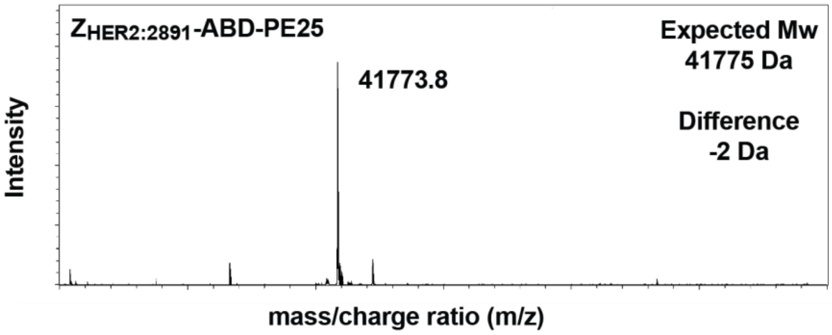

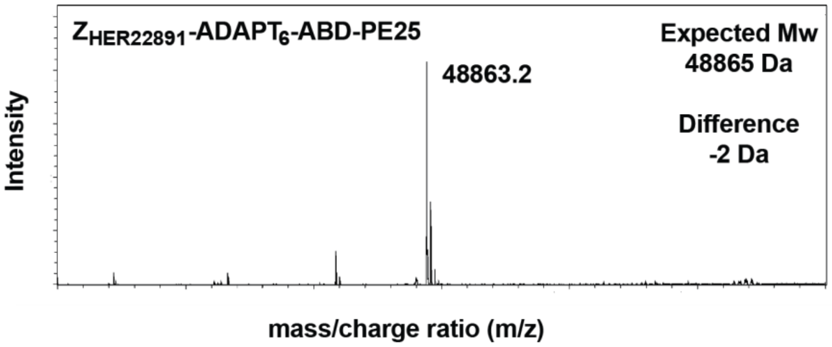

Figure S1. Analysis of molecular masses by mass spectrometry. Samples of the fusion toxins were analyzed by a Bruker impact II time of flight instrument fitted with an electrospray ionization source. The monoisotopic masses of the proteins were determined by deconvolution.

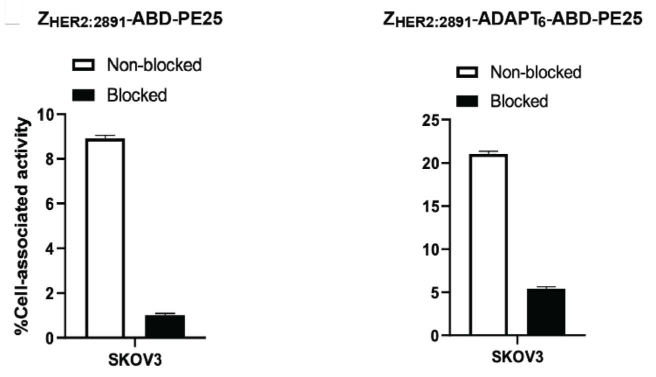

Figure S2. Specificity of binding. Fusion toxins, radiolabeled with ^{99m}Tc, were incubated with SKOV3 cells, that had been pre-incubated (blocked) or not pre-incubated (non-blocked) with the same fusion toxin without radiolabel. The cells were washed with PBS, followed by measurement of cell associated radioactivity. The panels show plots of the cell associated radioactivity as percentage of the activity of the added fusion toxin.
